# Reflections on major epidemics in history reported by online English news media and literature: interaction between epidemics and social conditions

**DOI:** 10.3389/fpubh.2023.1160756

**Published:** 2023-04-11

**Authors:** Xiaorui Chen, Xinyu Wei, Yuanfeng Zhang, Ying Sun, Zihan Xu, Shuyuan Zhang, Lin Li

**Affiliations:** ^1^Foreign Languages Teaching Department, School of International Studies, Binzhou Medical University, Yantai, China; ^2^The Second School of Clinical Medicine, Binzhou Medical University, Yantai, China; ^3^Medicine and Pharmacy Research Center, Binzhou Medical University, Yantai, China

**Keywords:** major epidemics, COVID-19 pandemic, social conditions, online English news media and literature, epidemic prevention and control, epidemic spread

## 1. Introduction

In the long course of history, human beings have been afflicted by various epidemics, infectious diseases and plagues. Pestis, smallpox, cholera, influenza, Ebola and so on existed or still exist in human society and have a huge impact on the development of society and the historical progress. The COVID-19 pandemic since 2020 has made the entire world different from the past in many aspects, including people's psychology, lifestyles, learning and teaching styles, etc. Meanwhile, social conditions also exert their effects on the spread of an epidemic, as well as on prevention and control. To control any potential follow-up pandemic and prevent the public from again experiencing a severe and widespread infection, it is important to study the interaction between epidemics and social conditions.

In this study, text-based data on historic major epidemics were mainly collected from online English news media reports and literature published from 2003 to 2023. Text-based artifacts refer to already existing documents, archives, or reports (1). They are commonly used as sources of evidence in qualitative data collection concerning policy studies and text studies (2). The content analysis method was employed to analyze the interaction between epidemics and social conditions, which includes the impact of major epidemics on human society and the impact of social conditions on major epidemics. This analysis may raise the public awareness of epidemic prevention and control. Additionally, public health workers and government agencies might be inspired by the understanding of the interaction between epidemic spread and social conditions, thereby acting to improve their response capacity in a future public health emergency.

## 2. The impact of major epidemics on human society

### 2.1. The impact of major epidemics on population and social psychology

The most immediate social impact of major epidemics is a massive reduction in population. Different diseases affect population size differently according to social periods. The Justinian Plague of 541–542 AD claimed nearly 100 million lives; The Black Death killed 50 million people between 1,346 and 1,350; The Spanish Influenza Pandemic of 1918–1919 claimed about 50 million lives around the globe (3); The COVID-19 pandemic caused more than 6 million people to deaths between 2020 and 2022, and the number is still increasing (4). The reason for the high fatality rate of epidemics is that they are highly contagious diseases and spread rapidly in a short period of time. In modern times, when overall medical care is better, fewer deaths have occurred than in the aforementioned plagues, but major epidemics have still dealt a large blow to human life. That is why the outbreak of a major epidemic causes social panic to some extent, and the degree of panic is related to social conditions at the time. During the reign of the Black Death, Europe was a society that was religious at its core, lacking basic medical care or an understanding of the disease's origin. Such social conditions led to widespread panic. Some Christian people regarded the Black Death as punishment from heaven because they believed they lived a life of sin (5), while others accused the Jews of poisoning wells, and hence thousands of Jews were killed as a result of this belief. Thus it can be seen that people are prone to act impulsively under the panic induced by epidemics, compounding the social crisis. At the beginning of COVID-19, episodes of so-called “panic buying” appeared in Canada due to fears about the rapid spread and potential deadliness of the novel coronavirus. However, excessive stockpiling of food and other supplies during the new coronavirus outbreak could actually be self-defeating, while accelerating the spread of the disease (6).

### 2.2. The impact of major epidemics on social life

The impact of major epidemics, especially the COVID-19 pandemic, are embodied in all walks of life. From the perspective of food, clothing, housing and transportation, the continuing epidemic has accelerated the process of establishing an e-world. As social distancing becomes the new normal of life, consumers are shifting from physical stores to online platforms for clothing, food, daily necessities, and healthcare, mainly through the use of various apps. At the same time, the rapid development of digital technology has created a variety of new business models. Accordingly, some countries have issued relevant policies to develop online medical services, propel telecommuting, accelerate the digital transformation of traditional enterprises, and build virtual industrial parks to promote the development of a shared economy (7). In terms of entertainment, the continuing COVID-19 pandemic has led to the suspension of offline tourism and the closure of many public entertainment venues such as cinemas. Therefore, cloud viewing, virtual tourism and virtual e-sports have become means of entertainment and relaxation for people (8, 9), trends in line with the current practice of maintaining social distance. Thus it can be seen that the degree of Internet use is increasing and social contact becomes gradually more virtual under the influence of the epidemic.

### 2.3. The impact of major epidemics on government agencies

In periods of epidemics, the most significant change in government agencies is the adjustment in working mechanisms and foci. During the COVID-19 pandemic, government agencies have piloted flexible working arrangements while advocating for employees working from home. This not only ensures the normal operation of government agencies but also provides convenient conditions for epidemic prevention and control, as well as travel by staff to promote consumption. This working mechanism may be a future trend due to a more highly developed Internet. In response to the pandemic, governments' foci have changed dramatically. Looking back at major outbreaks in history, such as cholera, SARS, MERS and now COVID-19, government agencies have concentrated on fighting the epidemic while simultaneously resuming work and production. In the fight against the COVID-19 epidemic, the focus of Chinese government agencies has mainly centered on initiating emergency public event responses, controlling population flow, avoiding mass gatherings and mobilizing medical resources (10). In terms of resumption of work and production, government agencies have carried out effective interventions, one of the typical characteristics of which is an increase in financial expenditures by government agencies (11).

By contrast, institutional dismantling or budget cuts have occurred in some countries or regions. Take Brazil for example: the central government dismantled the Brazilian Ministry of Agrarian Development and subsequently the National Secretariat for Food and Nutritional Security. The decentralization of the Brazilian National System for Food and Nutritional Security further led to budget cuts and administrative changes for key projects (12). As another example, Hong Kong was confronted with difficulties in formulating a budget for 2023 due to supply-chain disruptions, high inflation, tightened monetary policies, rising interest rates, and a contraction of the local economy partly caused by repercussions from the COVID-19 pandemic (13).

### 2.4. The impact of major epidemics on humanism

Major epidemics have a significant impact on the process of humanistic development. First of all, such an impact is reflected in education. Due to changes resulting from the COVID-19 pandemic, teachers and students have greater autonomy and choice. The traditional offline teaching mode has been transformed into an online teaching approach, and students have more autonomy in choosing their favorite courses and teaching methods rather than being constrained by having to attend on-campus courses. For instance, in the online mode, “nutritional knowledge” contrasted with “lack-of-nutrition knowledge” provides an example of a revolution in changing teaching and learning trends, impelled by students' options in the education field (14). In addition, the epidemic will completely change the traditional educational evaluation system. Students can directly give feedback to teachers and schools, and the hierarchy between teachers and students will be diluted (15). There are social media apps that allow students to report directly to teachers on how well they are doing, and schools ask students for feedback. This educational revolution endows teachers and students with rights, new freedoms and equality, thereby promoting humanistic development by affording them more flexibility.

The impact of the pandemic on humanistic progress has also increased in the speed of globalization. For a certain period of time, epidemics hindered people's communication in space and affected economic development. For instance, international businesses such as transport, oil and gas, extractive industries, and health care in the most affected countries of Sierra Leone, Liberia and Guinea were affected to varying degrees by Ebola. However, not only will this set of circumstances not end globalization, it will call for a new era of globalization with more humanistic caring while highlighting the global significance of the concept of a community with a shared future for mankind. Given the impact of the COVID-19 pandemic, countries around the globe have been providing mutual aid, maintaining reciprocal exchange, and sharing knowledge and research results, all of which undoubtedly play a positive role in awakening humanistic spirit and promoting global collaboration (16, 17).

## 3. The impact of social conditions on major epidemics

### 3.1. The impact of road traffic networks on epidemic spread

Influenza A (H1N1) reached several cities in mainland China in 2009 by means of imported cases (18) and spread as people traveled within China, primarily *via* airways, highways, and railways (19). As to the COVID-19 epidemic, rapid transportation has indeed increased the speed and scale of the spread of the epidemic. Dock workers along sea routes once became extremely high-risk groups. Since there are in the transportation, storage and sale of cold chain products, imported cases are still the top priority in China's epidemic prevention efforts. Concerning land transportation, the rapid development of express industry has brought greater convenience to people's lives. However, once the products sold online are infected, the related articles would be quickly sent to different places *via* express delivery. There is no denying that complex transport networks increase the risk of infection, despite the fact that a high flow of people are the root cause. This can be seen both in domestic news about traffic restrictions in severely affected areas and in foreign journals regarding transport policies introduced during the pandemic. That is why travel was restricted in nations and across nations (20).

### 3.2. The impact of socio-economic factors on epidemic spread

According to the report on SARS in 2003, the negative impact of SARS on the economy of China and Asia was clear. Tourism bore the brunt, followed by trade and investment (21). Epidemics have an impact on the economy, while the level of economic development also affects the spread of epidemics. Countries and regions with better economic environments have sufficient financial support and therefore are more likely to control the spread of epidemics to a certain extent. Take Canada for example: it invested a considerable amount of capital in vaccine production and led world in its vaccinated population. In slightly more than 2 months, after posting a record of more than 9,000 COVID-19 cases in a single day, Canada's daily case count shrank by 90% (22). Meanwhile, it should be noted that higher numbers of infection are recorded for groups that have higher population density, groups that have a higher proportion of youth, and groups that have lower income. Northeast Calgary set a clear example for this. According to the 2015 census, median household income in Ward 5 and Ward 10 was much less than the city-wide median; a comparably high percentage of households there have five or more people per home and both wards skew younger than the citywide average; infected residents there are working-class Calgarians and they work on the front lines (23). Therefore, socio-economic conditions were likely contributors to increased coronavirus rates in this city.

### 3.3. The role of modern technology in epidemic prevention and control

Artificial intelligence (AI) has already been employed to screen people to assess the risk of infection. For example, China deployed AI-powered temperature screening in public places during the COVID-19 pandemic. Temperature screening helps to detect symptoms and isolate suspected infections. Besides, thermal cameras have been adopted in COVID-19 case detection to quickly and accurately provide thermal imaging for body temperature. In addition, an AI-powered smartphone app was developed to track the geographical spread of the coronavirus, which aims to predict the population and communities who are the most vulnerable. This app also enables healthcare providers to disseminate information in real time while notifying individuals of potential infection hotspots in real time to avoid travel to those areas (24).

Big data plays an important role in tracing the source of infection and determining the movement trends of infected people. Such data can control close contacts in a short time and reduce the further spread of epidemics. Big data played a significant role during the COVID-19 outbreak. On June 11, 2020, a new case was reported in Xicheng District, Beijing. Since the infected person used a mobile phone to make a payment, the expert team employed big data analysis to quickly locate the source of the infection. This information was shared with other relevant departments, thus preventing the spread of the epidemic. This undoubtedly reflects the important role of the application of big data in determining the source of infection and controlling close contacts in a brief amount of time (25).

Big data can provide real-time monitoring of epidemic outbreaks, making epidemic prevention and control more efficient and convenient (26). As early as the Chikungunya outbreak in Europe in 2017, big data was used to assess the risk of virus transmission, virus import, and close dispersal of epidemic sources (27). Compared to previous outbreaks, the use of big data in the current surveillance of COVID-19 is unprecedented, with its open data set containing daily numbers of new infections by country (and even, in some cases, by city) (28). Dynamic data management and real-time information sharing have been fully realized. In addition, data query, statistics, and summaries are implemented according to different permissions.

During the COVID-19 epidemic, telemedicine has served as the first line of defense, employed by doctors to slow the spread of the coronavirus. Online consultations, telemonitoring, sensors and chatbots, etc., have reduced the time required for diagnosis and treatment. This advancement allows for rapid follow-up while allotting medical resources to various locations, thereby preventing the risk of contagion *via* professionals by avoiding direct physical contact and reducing the risk of exposure to respiratory droplets. An additional benefit of this advancement is an acceleration in the time required to train health professionals. Therefore, telemedicine based on the use of the Internet and associated technologies provides increased convenience and ready accessibility to information and communication related to health (29).

## 4. Conclusion

Based on online English news media and literature, this study investigated and analyzed major epidemics in history to explore the interaction between epidemics and social conditions from multiple perspectives. The findings show that major epidemics exert both positive and negative effects on society. On one hand, major epidemics had impact on population size, resulting in social panic, and meanwhile might lead to budget and institutional dismantlement of government agencies. On the other hand, major epidemics accelerated the progress of establishing an e-world in social life and education, promoted globalization with increased humanistic care and collaboration, and forced government agencies to change working mechanisms and foci on preparation for future pandemics. The findings also show that social conditions play a significant role regarding epidemics. Advanced road traffic networks increase the spread and scale of epidemics. Therefore, restrictions on population circulation represent a critical measure in epidemic prevention and control. Meanwhile, decreasing population density and improving economic conditions are also necessities. Additionally, modern technology such as AI, big data and telemedicine play an essential role in epidemic prevention and control. Hence, promoting the development of modern technology in an ongoing manner is the key to dealing with major epidemics in the future. Of course, all government agencies should perform their respective duties, and people should actively respond to minimize damage. A series of political and medical measures can be enacted to ease social and economic pressures. Rapid response, timely formulation of corresponding measures, and the establishment of specialized institutions for thorough elimination and prevention of disease following an outbreak can effectively alleviate or even end an epidemic. In future studies, it will be important to summarize the similarities and identify the differences among various epidemics. Only by learning lessons from history and developing a series of plans to face future emergencies can epidemics be controlled in a timely manner at the beginning of a given outbreak.

## Author contributions

XC conceived the idea, revised the initial manuscript in Chinese, and prepared the first draft in English. XW, YZ, YS, ZX, and SZ sourced the study data from online English news media and literature and analyzed the data and prepared the initial manuscript in Chinese. LL conceived the idea, revised the first draft in English, and provided extra materials for inclusion in the paper. XC and LL contributed to the final manuscript. All authors agree to be accountable for the content of the work.
